# Role of Gut Microbiota in Overweight Susceptibility in an Adult Population in Italy

**DOI:** 10.3390/nu15132834

**Published:** 2023-06-21

**Authors:** Cristina Politi, Marco Mobrici, Rosa Maria Parlongo, Belinda Spoto, Giovanni Tripepi, Patrizia Pizzini, Sebastiano Cutrupi, Daniele Franco, Renato Tino, Giuseppe Farruggio, Chiara Failla, Flavia Marino, Giovanni Pioggia, Alessandra Testa

**Affiliations:** 1 Institute of Clinical Physiology (IFC), National Research Council of Italy (CNR), 89124 Reggio Calabria, RC, Italy; politicristina89@gmail.com (C.P.); marco.mobrici88@gmail.com (M.M.); rosamariateresa.parlongo@cnr.it (R.M.P.); belinda.spoto@tin.it (B.S.); giovanniluigi.tripepi@cnr.it (G.T.); patrizia.pizzini@cnr.it (P.P.); sebastiano.cutrupi@cnr.it (S.C.); 2 Medilink S.r.l., Via Parma 36/A, Città Giardino, 96010 Melilli, SR, Italy; d.franco@medilink.it (D.F.); r.tino@medilink.it (R.T.); 3 SB SETEC S.p.A., Via Benedetto Croce 11, Città Giardino, 96010 Melilli, SR, Italy; giuseppe.farruggio@sbsetec.com; 4 Institute for Biomedical Research and Innovation (IRIB), National Research Council of Italy (CNR), 98164 Messina, ME, Italy; chiara.failla@irib.cnr.it (C.F.); flavia.marino@irib.cnr.it (F.M.); giovanni.pioggia@irib.cnr.it (G.P.); 5 Classical Linguistic Studies and Education Department, Kore University of Enna, 94100 Enna, EN, Italy

**Keywords:** gut microbiota, *Firmicutes*/*Bacteroidetes* ratio, overweight susceptibility, BMI, southern Italy population

## Abstract

Although the gut microbiota is known to affect body weight, its relationship with overweight/obesity is unclear. Our aim was to characterize microbiota composition in a cohort from the southernmost area of Italy. We investigated whether an altered gut microbiota could play an etiological role in the pathogenesis of overweight/obesity. A total of 163 healthy adults were enrolled. Microbiome analysis was performed via 16S rRNA gene sequencing. We found significant phylum variations between overweight (N = 88) and normal-weight (N = 75) subjects. *Bacteroidetes* and *Proteobacteria* were higher in overweight participants (*p* = 0.004; *p* = 0.03), and *Firmicutes* and *Verrucomicrobia* were lower (*p* = 0.02; *p* = 0.008) compared to normal-weight participants. Additionally, *Akkermansia* and *Bifidobacterium* (genus level) were significantly lower in the overweight group, as well as *Akkermansia muciniphila* at the species level. The *Firmicutes*/*Bacteroidetes* ratio (F/B ratio), an index of dysbiosis, was found to be inversely associated with BMI in linear and logistic regression models (*p* = 0.001; *p* = 0.005). The association remained statistically significant after adjustment for potential confounders. This cross-sectional study contributes to defining the gut microbiota composition in an adult population living in southern Italy. It confirms the relationship between overweight susceptibility and the dysbiosis status, highlighting the possible etiological role of the F/B ratio in disease susceptibility.

## 1. Introduction

In humans, the gastrointestinal tract is inhabited by several microorganisms (bacteria, yeasts and viruses) which establish a symbiotic relationship with the human host, and thus play a role in the physiological mechanisms that maintain a healthy organism [1]. Although it is not possible to exactly define the composition of a healthy human gut microbial [2,3], it is well known that *Firmicutes* and *Bacteroidetes* phyla are dominant and represent 90% of the whole gut community [4,5]. In fact, the ratio between these two phyla (commonly known as the *Firmicutes*/*Bacteroidetes* ratio or the F/B ratio) is used as an index to describe the fecal microbial composition [6]. A recent study published in the *Nature* portfolio showed that these two phyla and their ratio are a typical “scaffold” of a microbiologically healthy gut and, more generally, a healthy organism [7]. The gut microbiota impacts on human wellbeing through the regulation of the host metabolism, physiology, nutrition and immune function [8]. Specifically, it can be defined as a metabolic organ because, by providing enzymes that are not encoded by the human genome, it mediates the absorption of indigestible dietary polysaccharides and the synthesis of vitamins and essential amino acids [8,9]. This ability to generate energy from food and regulate the fatty acid composition [10,11] explains the scientific interest in establishing the existence of a relationship between a dysbiosis status (an imbalance of gut microbiota composition) and obesity [12,13]. Variation in the F/B ratio has been associated with demographic factors such as age and gender [14,15]. A huge number of animal and human studies have found a link between the F/B ratio and body weight, suggesting its potential usefulness as a biomarker of obesity susceptibility [16,17]. However, the F/B ratio trend in relation to the obesity status is still unclear. Some studies state that a high F/B ratio plays a key role in obesity susceptibility [18,19]. Others claim the opposite [12,13,16,18,19,20,21] and, finally, some studies find no significant association [22,23,24]. Several factors, such as age, gender, interaction with other phyla (i.e., *Proteobacteria*), environmental/genetic features, ethnicity and geographical origin of the study population may explain these controversial results [14,25,26]. Only considering studies in Italy, to avoid variations due to different geographical origins, we found only two research groups that had investigated the association of the F/B ratio with the overweight/obesity status and, once again, their results are conflicting [19,24].

We therefore investigated the relationship between the gut microbiota composition and overweight/obesity in a group of healthy adult subjects in southern Italy. Above all, we aimed to understand whether a state of gut dysbiosis (assessed by the F/B ratio) could play an etiological role in the pathogenesis of overweight/obesity.

## 2. Materials and Methods

### 2.1. Study Protocol and Participants

This observational study is a secondary analysis on a cohort of 163 healthy adults (>18 years of age), industrial workers, all residents of the area of Siracusa, Sicily. The study protocol was approved by the ethics committee of the University Hospital of Palermo (N°1/2021) and all participants signed an informed consent, written in accordance with the Declaration of Helsinki. At the same time as the recruitment, each participant reported their age, gender, weight, height, comorbidities, therapies and eating habits through a questionnaire. The questionnaire’s details are reported in Supplementary Materials (File S1). The participants were also explicitly asked to describe the following: diet (such as vegetarian, vegan), food intolerances, supplements (such as vitamins and probiotics), concomitant infections, and related antibiotic intake. The exclusion criteria were: pregnancy, transplantation, chronic disease, end-stage disease, and a known history of inflammatory bowel disease.

### 2.2. Laboratory Measurements

Blood sampling was performed in the early morning after an overnight fast. Serum bilirubin, cortisol, ferritin, transferrin and uricemia were measured using standard methods in a routine clinical laboratory. Vitamins (A, C and E) were assessed using the HPLC method (Chromsystem, Gräfelfing, Germany). Specific immunoenzymatic ELISA assays were used for the quantification of oxidized low-density lipoprotein (OxLDL), 3-nitrotyrosine (3-NT), advanced glycation end-products (AGEs), malondialdehyde (MDA), catalase (CAT), glutathione peroxidase 1 (GPX1), advanced oxidation protein products (AOPP), deoxyguanosine (8-OHdG), superoxide dismutase 2 (SOD2) and 4-hydroxynonenal (4-HNE) in the plasma or serum, according to the manufacturer’s instructions.

### 2.3. Faecal Sample Collection, DNA Extraction and 16S rRNA Sequencing

A fecal collection kit (OMNIgene^®^•GUT, OM-200, DNA Genotek Inc., Stittsville, ON, Canada) and its instructions for use were given to each participant. After collection, the stool samples were stored at room temperature, as indicated by the manufacturer. DNA extraction was performed using about 200 µL of fecal sample, employing a QIAamp Fast DNA Stool Mini Kit (Qiagen^®^, Hilden, Germany), according to the manufacturer’s instructions. The DNA concentration was evaluated using the Qubit fluorimeter with the dsDNA high-sensitivity (HS) assay kit (Invitrogen Q32854, Waltham, MA, USA), and later, the DNA yield was normalized to 1 ng/µL. Microbiome analyses were performed by sequencing the hypervariable regions V3-V4-V6 of the 16S rRNA gene through next-generation sequencing (NGS) technology. Amplicon libraries of the 16S regions were generated using a Microbiota Solution B kit (Arrow Diagnostics s.r.l., AD-002.024, Genoa, Italy). The amplification of the target regions, also known as PCR (polymerase chain reaction) target was performed by combining 15 µL of standard amp mix and 5 µL of DNA (1 ng/µL), whereas a second PCR (PCR index) was performed using a unique barcode per sample through the standard primers. Both reactions were performed in Mastercycler X50s (Eppendorf SE, Germany), the PCR product length was verified by 1.8% agarose gel electrophoresis and the amplicons purified with AMPure Beads XP (cat. n. A63880, Beckman Coulter, Inc., Brea, CA, USA). The libraries, normalized to a concentration of 4 nM were processed using a MiSeq Reagent Nano Kit v2 (500-cycles) (cat. n: MS-103-1003, Illumina, San Diego, CA, USA), following the “Denature and Dilute Libraries Guide-MiSeq system” of Illumina. The pooled library (3.5 pM) was corrected by adding 10% of Phix Control (cat. n. FC-110-3001, Illumina Inc.) at the same concentration (3.5 pM). The sequencing on MiSeq was performed using a MiSeqv2 Reagent kit. Raw data sequences were processed with MicrobAT (SmartSeq S.r.l., Novara, Italy).

### 2.4. Statistical Analyses

Descriptive statistics were carried out for each variable, using frequencies and percentages for categorical variables, the mean ± standard deviation (SD) for normally distributed data or median and interquartile range (IQR) for non-normally distributed data. The normal distribution of quantitative variables was assessed using the Kolmogorov–Smirnov test. Correlation analyses were performed with the Pearson product moment correlation coefficient (r) and *p*-value. Grubb’s test was applied to identify the potential outliers.

The etiological association between the F/B ratio and body mass index (BMI) was investigated using linear (when considering BMI as continuous variable) and logistic (when considering BMI as categorical variable, i.e., normal overweight/obesity versus normal weight) regression analyses of increasing complexity. In detail, from models 2 to 5 of the multivariable regression analyses, a series of potential confounders was included, i.e., variables which were associated with *p* ≤ 0.10 to both the F/B ratio and BMI. Furthermore, we replicated the linear regression analysis by using the waist circumference (WC) as a dependent variable. The WC was estimated using a validated model described by Bozeman et al. [27]. To understand whether the bioactive compounds of specific nutrients (i.e., yogurt, fruits/vegetables and whole foods) mediated the F/B ratio–BMI relationship, these three variables were simultaneously included in the model 6. A reduction in the strength of the F/B ratio–BMI link was interpreted as a mediation effect. In linear regression models, data were expressed as standardized regression coefficients (β) and *p*-values (statistical significance *p* ≤ 0.05). In logistic regression models, data were expressed as an odds ratio, 95% CI, and *p*-value. Data analysis was performed using v. 19 of SPSS for Windows (Chicago, IL, USA).

## 3. Results

### 3.1. Study Cohort

This observational study included 163 healthy adults recruited in Syracusa, Sicily (Italy). The main demographic, somatometric and clinical data of the study group are reported in Table 1, along with comorbidities. The mean age of the participants was 47.5 years, 73% were males and 16% were smokers. The percentage of overweight subjects (BMI ≥ 25 kg/m^2^) in our population was quite high (54.0%) compared to the prevalence observed in the adult population in Italy (46%), according to the 4th Italian Obesity Barometer Report 2022 [28].

**Figure 1:**
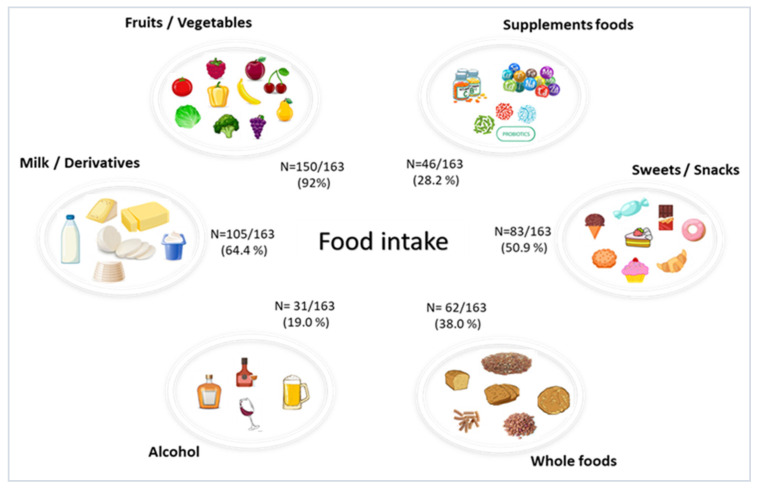
Average frequency of daily intake of specific food groups in the cohort of the study data are expressed as n (%).

Overall, the eating habits of the study cohort were in line with a Mediterranean diet, with a high consumption of fruits, vegetables and whole foods (Figure 1). No particular diet, such as vegetarian or vegan, was indicated by the participants. The measured analytes and oxidative stress markers are shown in Table 2.

### 3.2. Gut Microbiota Composition of the Study Cohort

**Figure 2:**
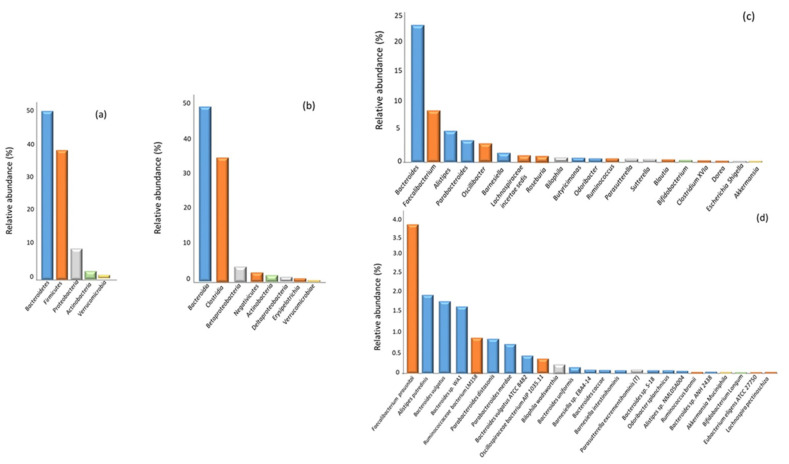
Distribution of the gut microbiome composition in the study population (n = 163) at different rank levels: (**a**) phylum, (**b**) class, (**c**) genus and (**d**) species. Data are expressed as a relative abundance. Only the bacteria with a relative abundance value ≥0.1% are considered.

The gut microbiota composition of our population is shown in Figure 2. The bacterial frequencies are expressed in terms of relative abundance at the different rank levels of the phylum, class, genus and species. As expected, the *Bacteroidetes* and *Firmicutes* were the most frequent phyla in the rank (mean value 49.4 ± 9.20% and 38.1 ± 9.56%, respectively), followed by *Proteobacteria* with 6.05% (3.95–9.34), *Actinobacteria* with 0.91% (0.32–2.24) and *Verrucomicrobia* with 0.06% (0–1.76). The mean value of the *Firmicutes*/*Bacteroidetes* ratio (F/B ratio) was 0.83 ± 0.36.

In order to evaluate whether there were differences in the gut microbiota composition according to the BMI, we divided the population according to the BMI (cut-off value ≥ 25 kg/m^2^). The relative phyla abundance was significantly different between the two groups (overweight/obese vs. normal weight): *Bacteroidetes* (51.3 ± 8.8% vs. 47.2 ± 9.2%, *p* = 0.004), *Firmicutes* (36.5 ± 9.3% vs. 39.9 ± 9.6%, *p* = 0.02), *Proteobacteria* (7.25%, 4.25–9.98 vs. 5.35%, 3.65–8.04; *p* = 0.03) and *Verrucomicrobia* (0.01%, 0.00–1.12 vs. 0.84%, 0.00–2.45; *p* = 0.008). No difference was found for *Actinobacteria* (0.87%, 0.29–1.65 vs. 0.94%, 0.36–3.13; *p* = 0.83). In terms of lower taxonomic levels, only *Akkermansia* and *Bifidobacterium* (genus level), and *Akkermansia muciniphila* (species level) were lower in the overweight than in normal-weight subjects, with significant values of *p* = 0.038 (0.03%, 0.01–0.70 vs. 0.43%, 0.01–2.02), *p* = 0.036 (0.21%, 0.05–0.72 vs. 0.33%, 0.08–1.55) and *p* = 0.02 (0.02%, 0.00–0.63 vs. 0.38%, 0.01–1.71; *p* = 0.02), respectively. Table S1 summarizes the median values of the phyla, families, classes, genera and species with abundance ≥0.1%.

### 3.3. Relationship between the Gut Microbiota Composition and Overweight/Obesity

A total of 7,055,425 sequences were obtained from 163 samples, with a median value of 43,306 (IQR: 31,372–53,965) reads per sample. The total reads assigned to taxa by the MicrobAT software were 6,033,218, with reads per sample equal to 36,426 (IQR: 28,497–44,800). First, the relationship between the gut microbiota composition and overweight/obesity was investigated by a univariate analysis between the F/B ratio and BMI, either by considering the BMI as a continuous variable or as a binary variable.

**Figure 3:**
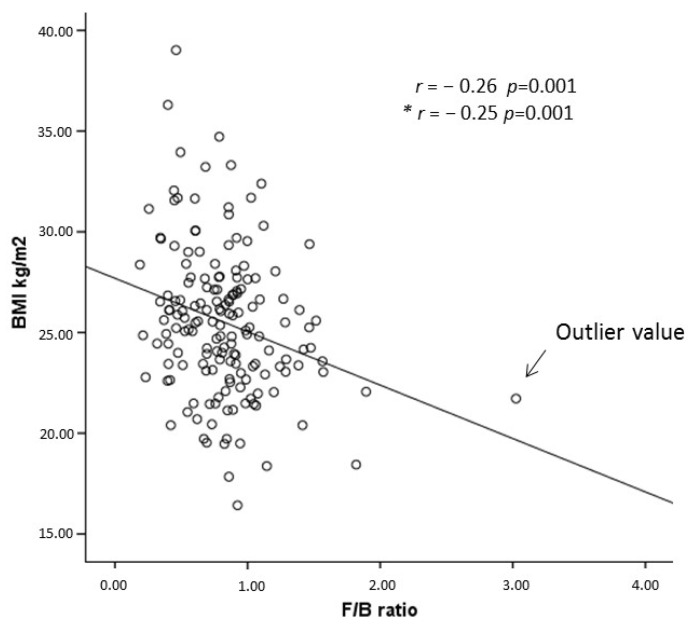
Correlation analysis scatter plot between the Firmicutes/Bacteroidetes ratio (F/B ratio) and Body Mass Index (BMI). The results are expressed as a correlation coefficient (r) and *p*-value (*p*). (*) Results obtained excluding the outlier (F/B ratio = 3.02; BMI = 21.7 kg/m^2^).

As shown in the scatter plot in Figure 3, the F/B ratio was found to be inversely related to the BMI (r = −0.26, *p* = 0.001). This correlation remained unchanged after the exclusion of the outlier (F/B ratio = 3.02; BMI = 21.7 kg/m^2^) (r = −0.25, *p* = 0.001). In line with this observation, a one-unit increase in the F/B ratio was associated with a 76% lower odds ratio of overweight/obesity (OR: 0.24, 95% CI: 0.06–0.99, *p* = 0.005) (See Table 4 in Section 3.4).

### 3.4. Univariable and Multivariable Regression Analyses of the Relationship between the F/B Ratio and BMI

In order to investigate the etiological role of the microbiota composition, as assessed by the F/B ratio, in the pathogenesis of the overweight subjects, linear and logistic regression models of increasing complexity were created (Models 1–6, Table 3 and Table 4). The association between the F/B ratio and BMI remained statistically significant after adjustment for demographic and clinical data (Model 2, Table 3), the Shannon index (the diversity microbial index), as well as for phyla (Model 3, Table 3), classes/genera (Model 4, Table 3) and species (Model 5, Table 3).

Notably, when yogurt intake and fibre consumption (as assessed by the fruit/vegetable and whole food intake) were jointly introduced into the model (Model 6, Table 3), the strength of the F/B ratio–BMI relationship decreased drastically (β from −0.18 to −0.12, −33%) and generally became non-significant. The same analysis was performed with waist circumference as a dependent variable, and the association was completely confirmed (Table S2). A multivariable logistic regression analysis of increasing complexity, with overweight as the dependent variable, provided similar results (Table 4). In fact, in this analysis, data adjustment for potential confounders, except for yogurt and fibre intake, also did not change the strength of the association between the F/B ratio and overweight (Model 1–5, Table 4). Closely parallel with the results of the linear multivariable modeling, the inclusion of yogurt and fibre intake (see Model 6 in Table 4) reduced the strength of the association between the F/B ratio and the outcome variable.

## 4. Discussion

Our study revealed that there is an independent relationship between the gut microbiota composition and overweight/obesity susceptibility in a population of healthy adults from southern Italy. *Firmicutes* and *Bacteroidetes* represent the most frequent phyla within the gut microbiota and in this study, the F/B ratio (an index of dysbiosis) was significantly and inversely related to the BMI and WC, also after adjustment for potential confounders. Furthermore, when yogurt intake and fibre consumption (as assessed by the fruit/vegetable and whole food intake) were jointly introduced into linear multivariable models, the strength of the F/B ratio–BMI relationship decreased drastically and generally became non-significant, suggesting that the intake of yogurt and fibres are involved in the causal pathway connecting the microbiota composition (F/B ratio) to overweight (BMI). We obtained the same results in logistic multivariable regression analysis, confirming that these foods could act as a mediator between microbiota and overweight.

In line with previous studies [21,29,30,31,32,33], we found an unbalanced F/B ratio, which is in favor of *Bacteroidetes* in the overweight/obese group. However, some results of animal and human studies are in contrast with our evidence, showing an inverse F/B ratio trend (higher relative abundance of *Firmicutes* than *Bacteroidetes*) in obese subjects, or no statistically significant differences compared to the normal-weight subjects [18,19,23,24,34,35].

The heterogeneity of the study population (age, gender, cultural and lifestyle habits, environmental factors, ethnicity) and/or the differences in experimental phases (sample processing, data analysis, type of sequencing platform, primers, inadequate statistical power) could explain these discrepancies [4,16,20,36]. In particular, the geographical origin of the study subjects is an important element influencing the gut composition, mainly due to the variability in dietary behavior [26,37]. Therefore, only considering the studies carried out in Italy, only one work reported the gut dysbiosis status significantly related to overweight. Unlike our results, the authors found a higher F/B ratio in obese subjects compared to the normal-weight subjects [19]. This difference could be due to the high prevalence of women in this latter study compared to our cohort (87% versus 27%). In fact, gender plays a key role in gut bacterial composition, with the *Bacteroidetes* abundance being significantly lower in women [38].

### Gut Microbiota–Obesity Association Hypothesis

Inflammation is known to be a hallmark of obesity [39], and is likely the driving force of the microbiota–obesity association [40]. However, the gut microbiota plays a key role in regulating intestinal immunity, causing local and systemic inflammation associated with obesity progression [41]. Evaluating gut microbiota differences according to the BMI, the overweight/obese subjects have been characterized by a pro-inflammatory gut microbiome profile: *Proteobacteria* and *Bacteroidetes* (phyla with pro-inflammatory properties) [42] were higher, whereas *Verrucomicrobia* (with anti-inflammatory properties) [43] were lower in overweight than in normal-weight subjects.

**Figure 4:**
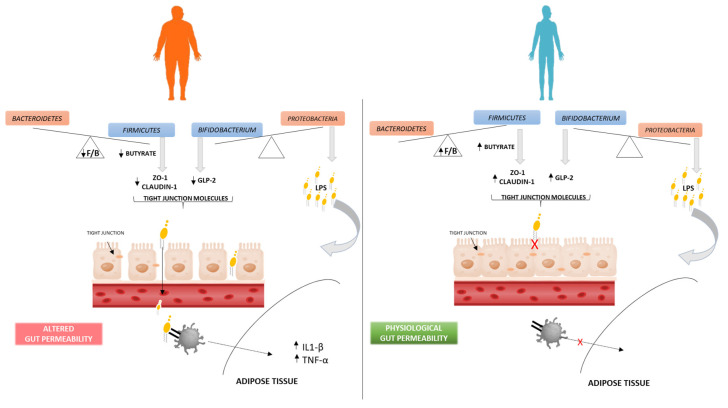
Gut microbiota–obesity association hypothesis. In overweight subjects (**left** panel), the low level of *Firmicutes* (low F/B ratio) and *Bifidobacterium* alter the production of the tight junction molecules. The lower level of *Firmicutes* leads to a low production of butyrate, derived from fermentation of dietary fibres, which then leads to a lower production of the ZO-1 and CLAUDIN-1 molecules. A low level of *Bifidobacterium* directly determines a low production of GLP-2. These events lead to an altered gut permeability, with LPS leakage (located in the outer membrane of *Proteobacteria*) from the intestinal lumen into the circulation, which induces an immune pro-inflammatory response in the overweight group compared to the normal-weight group (**right** panel). The circulating LPS, recognized by the TLR-4 receptor, promotes macrophage accumulation in adipose tissue, triggering the secretion of pro-inflammatory cytokines (IL-1β and TNF-α).

Modification of the gut barrier permeability, along with the release of lipopolysaccharides (LPSs) located in the outer membrane of *Proteobacteria* (Gram-negative bacteria), is a pathway through which the gut microbiota can affect obesity status [44]. In an animal model, Cani et al. demonstrated that obesity is related to a reduction in *Bifidobacterium* levels and consequently to the decreased production of GLP-2, a key molecule for maintaining the intestinal barrier integrity [45]. An altered gut microbiota leads to an impairment in intestinal permeability, which causes the leakage of bacterial antigens (such as LPS) from the intestinal lumen into the circulation, thus inducing an immune pro-inflammatory response in the host. Circulating LPSs migrate to different organs, such as adipose tissue, triggering an innate immune response. In detail, the complex LPS–LBP (LPS-binding proteins) binds the TLR-4 receptor on plasma macrophage surfaces, triggering a transduction signal that determines the expression of pro-inflammatory molecules (cytokines, growth factors, chemokines, adhesion molecules, etc.). Thanks to these events, the macrophages acquire the M1 phenotype (classically activated form) with characteristic pro-inflammatory properties. In fact, in adipose tissue, the M1 macrophages secrete high levels of pro-inflammatory cytokines (TNF-α, IL-6 and IL-1β), inducing an inflammation state and an impairment of insulin sensitivity, typically associated with obesity [44,46,47]. Studies in humans have demonstrated that an increase in serum LPS and LPS-binding proteins (LBPs) was associated with obesity [48]. In addition, subjects undergoing weight loss interventions via bariatric surgery showed an improvement in glucose tolerance and a reduction in both LPS and LBP concentrations [49]. In accordance with these observations, in our overweight subjects, a significant reduction in *Bifidobacterium* levels was observed, accompanied by an increase in the levels of *Proteobacteria*, holding LPS in its membrane (Figure 4). Once we had verified the existence of the F/B ratio–BMI relationship, we explored whether the F/B ratio played an etiological role in overweight susceptibility. In our logistic regression analysis, a one-unit increase in *Firmicutes*/*Bacteroidetes* ratio was associated with a 76% lower odds ratio of overweight/obesity, also after adjusting for potential confounders. In other words, the higher the F/B ratio, the lower the obesity risk.

In addition, through regression models of increasing complexity, we identified yogurt, vegetables/fruits and whole foods, processed by bacterial digestion, as mediators in this relationship. The fact that gut bacteria can produce short-chain fatty acids (SCFAs) through the fermentation of dietary fibers could explain the role of gut dysbiosis in obesity-related inflammation [44,50]. In particular, butyrate, which is mainly produced by gut bacteria belonging to *Firmicutes*, acts through different mechanisms, decreasing metabolism efficiency, improving fatty acid oxidation, reducing reactive oxygen species (ROS) generation, modulating mitochondrial function efficiency and then decreasing fat mass, inflammation and insulin resistance [51]. Furthermore, it prevents the transfer of bacterial endotoxins through the gut barrier [29]. Gonzalez et al. demonstrated that butyrate improves the integrity of the intestinal barrier, promoting the overexpression of two proteins involved in the tight junction formation (ZO-1 and claudin-1) [52]. This improvement in barrier integrity therefore leads to a significant decrease in LPS leakage, thereby reducing insulin resistance and the overall dysmetabolic function [52], as shown in Figure 4. Thus, this suggests the etiological and protective role of a high F/B ratio in overweight susceptibility.

There are some limitations of our study that are worth mentioning. Firstly, the cross-sectional nature of the study precludes us from delineating a time sequence and inferring causality relationships. However, the F/B ratio-BMI association is statistically robust, remaining significant both in linear and logistic regression models of increasing complexity. Secondly, we suggested the role of LPSs only considering the relative abundance of bacteria and not its circulating levels. Finally, we used a basic questionnaire regarding the eating habits of participants, without detailed information on the quality and the quantity of nutrient intakes. Likewise, the anthropometric data used to calculate the BMI and estimate the WC were self-reported.

## 5. Conclusions

This study contributes to defining the gut microbiota composition in a population in Italy. In addition, we confirmed a relationship between overweight/obesity susceptibility and the dysbiosis status, and highlighted the possible etiological role of the F/B ratio in the disease susceptibility. In a future perspective, given the importance of oxidative stress in obesity [53], we will conduct further analysis on the biomarkers of oxidative stress to learn more about their role in this condition and evaluate their relationship with the gut microbiota composition. However, further studies in a larger population are needed to confirm the association between dysbiosis status and overweight/obesity. Intervention studies should also be performed in obese patients that are following a strict diet to confirm that the F/B ratio is a potential biomarker of obesity susceptibility.

## Figures and Tables

**Table 1:** Demographic, somatometric and clinical data of the study group.

Demographic and Clinical Characteristics	N = 163
Age, years	47.5 ± 11.4
Male sex, n (%)	119 (73.0)
Height, cm	174 (167–178)
Weight, kg	76.3 ± 15.1
BMI, kg/m^2^	25.5 ± 3.70
BMI ≥ 25 kg/m^2^, n (%)	88 (54.0)
Waist Circumference, cm	93.2 ± 11
Smoker (y/n), n (%)	26 (16.0)
Physical activity (y/n), n (%)	92 (56.4)
**Comorbidities (y/n), n (%)**	
Cardiovascular diseases	7 (4.3)
Hypertension	30 (18.4)
Hypercholesterolemia	22 (13.5)
Diabetes	4 (2.5)
Thyroid diseases	16 (9.8)
Rheumatic diseases	8 (4.9)
Autoimmune diseases	4 (2.5)
Liver diseases	1 (0.6)
Respiratory diseases	25 (15.3)
Kidney diseases	5 (3.1)
Pharmacological therapies	40 (24.5)
Antibiotic therapies	10 (6.1)

Data are given as n (%), mean and standard deviation (SD), or as the median and interquartile range (IQR), as appropriate. BMI: Body Mass Index. y/n: yes/no.

**Table 2:** Dosage of analytes and oxidative stress markers in the study group.

Molecules	N = 163
Vitamin C, µmol/L	35.5 (23.9–55.2)
Vitamin A, µmoli/L	1.79 ± 0.42
Vitamin E, µmoli/L	31.1 ± 7.51
Transferrin, gr/L	2.58 (2.37–2.87)
Cortisol, nmol/L	312 (255–407)
Ferritin, ng/mL	62.6 (19.8–127)
Uricemia, mg/dl	5.10 ± 1.33
Bilirubin, mg/dl	0.69 (0.55–0.92)
8-iso-PGF2α, pg/mL	331 ± 136
3-NT, ng/mL	54.1 ± 20.7
AGEs, ng/mL	t7.82 (5.19–10.6)
MDA, ng/mL	126 (109–154)
CAT, pg/mL	179 (148–345)
OxLDL, ng/mL	38.4 (15.6–116)
GPX1, ng/mL	22.9 (17.4–30.8)
AOPP, ng/mL	0.49 (0.39–0.59)
8-OHdG, ng/mL	3.98 ± 0.75
SOD-2, ng/mL	7.72 (4.48–11.3)
4-HNE, pg/mL	11987 ± 5719

Data are given as the mean and standard deviation (SD), or as the median and interquartile range (IQR), as appropriate. 3-NT: 3-nitrotyrosine; AGE: advanced glycation end-products; MDA: malondialdehyde; CAT: catalase; OxLDL: oxidized low-density lipoprotein; GPX1: glutathione peroxidase 1; AOPP: advanced oxidation protein products; 8-OHdG: deoxyguanosine; SOD2: superoxide dismutase 2; 4-HNE: 4-hydroxynonenal.

**Table 3:** Univariable and multivariable linear regression analyses.

Dependent Variable: BMI	Model 1/Crude (Beta, *p*)	Model 2 (Beta, *p*)	Model 3 (Beta, *p*)	Model 4 (Beta, *p*)	Model 5 (Beta, *p*)	Model 6 (Beta, *p*)
*Firmicutes*/*Bacteroidetes* Ratio	−0.26 (0.001)	−0.26 (<0.001)	−0.19 (0.011)	−0.18 (0.031)	−0.18 (0.035)	−0.12 (0.154)
Age, yr		0.11 (0.106)	0.12 (0.095)	0.11 (0.133)	0.11 (0.128)	0.14 (0.055)
Male sex, (y/n)		−0.43 (<0.001)	−0.40 (<0.001)	−0.40 (<0.001)	−0.40 (<0.001)	−0.39 (<0.001)
Cardiovascular diseases (y,n)		0.16 (0.020)	0.16 (0.022)	0.17 (0.018)	0.17 (0.019)	0.14 (0.043)
Thyroid diseases (y,n)		0.05 (0.490)	0.07 (0.315)	0.07 (0.323)	0.07 (0.342)	0.09 (0.202)
Shannon Index			−0.06 (0.443)	−0.05 (0.517)	−0.05 (0.515)	−0.03 (0.664)
Phylum *Actinobacteria*			−0.005 (0.946)	0.03 (0.927)	0.03 (0.919)	0.05 (0.879)
Phylum *Proteobacteria*			0.07 (0.340)	0.04 (0.617)	0.04 (0.632)	0.04 (0.629)
Phylum *Verrucomicrobia*			−0.13 (0.076)	−0.45 (0.437)	−0.43 (0.470)	−0.23 (0.689)
Class *Actinobacteria*				0.04 (0.898)	0.04 (0.905)	0.001 (0.998)
Class *Verrucomicrobia*				−0.12 (0.982)	−0.005 (0.999)	0.14 (0.978)
Class *Betaproteobacteria*				0.03 (0.791)	0.03 (0.789)	0.02 (0.838)
Genus *Bifidobacterium*				−0.10 (0.387)	−0.10 (0.398)	−0.05 (0.653)
Genus *Akkermansia*				0.45 (0.936)	0.007 (0.999)	−0.50 (0.928)
Genus *Sutterella*				0.02 (0.862)	0.02 (0.856)	0.04 (0.660)
Species *Akkermansia muciniphila*					0.30 (0.741)	0.44 (0.618)
Yogurt intake, (y/n)						−0.08 (0.283)
Whole food intake, (y/n)						−0.12 (0.113)
Fruit and vegetale intake, (y/n)						−0.16 (0.021)

Data are expressed as linear regression coefficients and *p*-values. Dependent variable: BMI.

**Table 4:** Univariable and multivariable logistic regression analyses.

Dependent Variable: 25 < BMI ≥ 25	Model 1/Crude OR (95%, CI), *p*	Model 2 OR (95%, CI), *p*	Model 3 OR (95%, CI), *p*	Model 4 OR (95%, CI), *p*	Model 5 OR (95%, CI), *p*	Model 6 OR (95%, CI), *p*
*Firmicutes/Bacteroidetes* Ratio	0.24 (0.09–0.66), 0.005	0.18 (0.06–0.60), 0.005	0.25 (0.07–0.87), 0.030	0.23 (0.05–0.95), 0.042	0.24 (0.06–0.99), 0.049	0.39 (0.08–1.92), 0.25
Age, yr		1.03 (0.99–1.06), 0.076	1.03 (0.99–1.07), 0.072	1.02 (0.99–1.06), 0.209	1.02 (0.99–1.06), 0.203	1.03 (0.99–1.07), 0.128
Male sex, (y/n)		0.18 (0.07–0.42), <0.001	0.18 (0.08–0.44), <0.001	0.14 (0.05–0.38), <0.001	0.14 (0.05–0.37), 0.135	0.09 (0.03–0.29), <0.001
Cardiovascular diseases, (y/n)		10.8 (0.69–171), 0.090	10.6 (0.69–163), 0.091	45.1 (1.24–1634), 0.038	45.6 (1.25–1669), 0.037	154 (2.31–10,239), 0.019
Thyroid diseases, (y/n)		1.07 (0.30–3.77), 0.922	1.03 (0.28–3.86), 0.961	1.14 (0.28–4.73), 0.857	1.11 (0.27–4.62), 0.884	1.25 (0.27–5.67), 0.777
Shannon Index			0.73 (0.16–3.31), 0.684	0.97 (0.20–4.74), 0.967	0.95 (0.20–4.66), 0.952	1.22 (0.20–7.32), 0.828
Phylum *Actinobacteria*			1.02 (0.90–1.15), 0.762	84.9 (0.79–9145), 0.063	92.2 (0.87–9763), 0.057	200 (0.87–46,084), 0.056
Phylum *Proteobacteria*			1.04 (0.95–1.13), 0.404	1.00 (0.90–1.11), 0.959	1.00 (0.90–1.10), 0.934	1.00 (0.90–1.11), 0.947
Phylum *Verrucomicrobia*			0.98 (0.86–1.11), 0.749	0.43 (0.13–1.45), 0.175	0.45 (0.13–1.53), 0.202	0.45 (0.11–1.77), 0.253
Class *Actinobacteria*				0.071 (0.0–1.47), 0.072	0.01 (0.0–1.33), 0.066	0.006 (0.0–1.34), 0.064
Class *Verrucomicrobia*				0.001 (0.0–288), 0.293	0.002 (0.0–363), 0.308	0.002 (0.0–1122), 0.350
Class *Betaproteobacteria*				1.07 (0.85–1.34), 0.568	1.07 (0.85–1.35), 0.560	1.10 (0.86–1.41), 0.432
Genus *Bifidobacterium*				0.60 (0.37–0.97), 0.037	0.60 (0.38–0.97), 0.038	0.58 (0.35–0.98), 0.043
Genus *Akkermansia*				1693 (0.007–10^3^), 0.243	925 (0.003–10^3^), 0.294	713 (0.001–10^3^), 0.352
Genus *Sutterella*				0.95 (0.78–1.16), 0.601	0.95 (0.78–1.16), 0.614	0.96 (0.78–1.17), 0.675
Species *Akkermansia muciniphila*					1.54 (0.24–9.76), 0.648	2.10 (0.34–13.04), 0.427
Yogurt intake, (y/n)						0.26 (0.19–0.69), 0.005
Whole food intake, (y/n)						0.45 (0.18–1.09), 0.076
Fruit and vegetale intake, (y/n)						0.30 (0.05–1.89), 0.199

Data are expressed as an odds ratio (OR), 95% confidence interval (CI) and *p*-value. Dependent variable: 25 < BMI ≥ 25.

## Data Availability

Data are contained within the article and Supplementary Materials. The data presented in this study are available on request from the corresponding author. The data are not publicly available due to privacy issues.

## Supplementary Materials

The following supporting information can be downloaded at: https://www.mdpi.com/article/10.3390/nu15132834/s1, Table S1: Summary of the median values of the phyla, families, classes, genera and species with abundance ≥0.1% among the whole population and the population divided according to the Body Mass Index (BMI). Table S2: Univariable and multivariable linear regression analyses; dependent variable: waist circumference (WC).
