# Supplementary material for: Role of Gut Microbiota in Overweight Susceptibility in an Adult Population in Italy
Source: Nutrients. 2023 Jun 21;15(13):2834. doi: 10.3390/nu15132834 (PMC10343630; doi:10.3390/nu15132834)
Supplement: Supplementary file 1 [file nutrients-15-02834-s001.zip › File S1.pdf]

QUESTIONNAIRE

Anonymous code of participant: ……………

1. Age: ………………….
2. Gender: …………….
3. Height: ……………….
4. Weight: ………………
5. Are you affected by cardiovascular comorbidities? ☐ NO ☐ YES

   If yes which ones?..

   How long?...
6. Are you affected by hypertension? ☐ NO ☐ YES

   How long?...

   Are you in drug therapy? ....
7. Are you affected by hypercholesterolemia? ☐ NO ☐ YES

   How long?...

   Are you in drug therapy? ....
8. Are you affected by diabetes? ☐ NO ☐ YES

   Type I or type II diabetes?..

   Are you in drug therapy? ....
9. Are you affected by thyroid disease? ☐ NO ☐ YES

   Which ones?..

   How long? ....
10. Are you affected by rheumatic diseases (arthrosis/arthritis)? ☐ NO ☐ YES

    Which ones?..

    How long? ....
11. Are you affected by autoimmune diseases? ☐ NO ☐ YES

    Which ones?..

    How long? ....
12. Are you affected by gastritis, ulcer or irritable bowel disease? ☐ NO ☐ YES

    Which ones?..

    How long? ....

13. Are you affected by liver diseases? ☐ NO ☐ YES
Which ones?..
How long? ....

14. Are you affected by respiratory diseases (bronchitis, asthma, allergy)? ☐ NO ☐ YES
Which ones?..
How long? ....

15. Are you affected by kidney diseases? ☐ NO ☐ YES
Which ones?..
How long? ....

16. Have you recently (within one month) had gastrointestinal symptoms (such as diarrhea, vomit, constipation)? ☐ NO ☐ YES
How many?...

17. Have you recently (within one month) effectuated antibiotic therapies? ☐ NO ☐ YES
Which ones?..
How long? ....

18. Do you carry out continuative pharmacological therapies? ☐ NO ☐ YES
19. Have you been affected by childhood obesity? ☐ NO ☐ YES
20. Have you recently changed your eating habits (vegeterian, vegan, strict regiment diet)? ☐ NO ☐ YES
Which ones?..
How long? ....
21. Have you recently used supplements foods (such as vitamins, probiotics, fibres, mineral supplements, etc.)? ☐ NO ☐ YES
Which ones?..
How long? ....
22. Do you daily eat yogurt? ☐ NO ☐ YES
23. Do you daily eat integral foods and/or foods rich in fibres? ☐ NO ☐ YES
Which ones?..
24. Do you daily eat fruits and vegetables? ☐ NO ☐ YES
Which ones?..
25. Do you daily eat sweats and snacks? ☐ NO ☐ YES
Which ones?..
26. Do you daily eat milk and derivatives (cheeses)? ☐ NO ☐ YES
Which ones?..

27. Do you drink alcohol? ☐ NO ☐ YES

Which ones?...

How many (daily, weekly or monthly)?...

28. Are you an active smoker (currently smokes)? ☐ NO ☐ YES

29. Have you smoked in the past? ☐ NO ☐ YES

How long?..

30. Do you do physical activity? ☐ NO ☐ YES

Which one?...

How many (daily, weekly or monthly)?...
