# Supplementary material for: Role of Gut Microbiota in Overweight Susceptibility in an Adult Population in Italy
Source: Nutrients. 2023 Jun 21;15(13):2834. doi: 10.3390/nu15132834 (PMC10343630; doi:10.3390/nu15132834)
Supplement: Supplementary file 1 [file nutrients-15-02834-s001.zip › Table S1.pdf]

Table S1. Summary of the median values of phyla, classes, families, classes, genera and species with relative abundance ≥ 0.1%

	**Whole population (n=163)**	**BMI< 25 kg/m2 (n=75)**	**BMI ≥ 25 kg/m2 (n=88)**
**Phyla**			
Bacteroidetes	49.2% (44.2-55.1)	47.6% (40.9-53.5)	50.5% (45.5-58.1)
Firmicutes	39.3% (31.3-44.3)	41.6% (34.7-45.4)	37.8% (28.7-44.0)
Proteobacteria	6.05% (3.95-9.34)	5.35% (3.65-8.04)	7.25% (4.25-9.97)
Actinobacteria	0.91% (0.32-2.24)	0.94% (0.36-3.13)	0.87% (0.29-1.65)
Verrucomicrobia	0.10% (0.0 -1.76)	0.84% (0.00-2.45)	0.01%(0.00-1.12)
**Classes**			
Bacteroidia (P: Bacteroidetes)	48.2% (42.7-54.4)	46.2% (39.5-52.0)	49.4%(44.6-57.4)
Clostridia (P: Firmicutes)	36.5% (27.4-41.9)	37.8% (31.2-42.3)	34.6% (25.9-41.5)
Betaproteobacteria (P: Proteobacteria)	3.32% (1.82-4.91)	3.24% (1.64-4.67)	3.53% (1.85-5.32)
Negativicutes (P: Firmicutes)	1.25% (0.56-1.87)	1.14% (0.55-1.63)	1.31% (0.57-2.14)
Actinobacteria (P: Actinobacteria)	0.94% (0.36-2.21)	1.09% (0.45-3.13)	0.87% (0.29-1.63)
Deltaproteobacteria (P: Proteobacteria)	0.85% (0.44-1.46)	0.89% (0.49-1.46)	0.75% (0.38-1.45)
Gammaproteobacteria (P: Proteobacteria)	0.24% (0.04-1.10)	0.23% (0.00-0.78)	0.26% (0.06-1.63)
Erysipelotrichia (P: Firmicutes)	0.34% (0.19-0.58)	0.41% (0.21-0.74)	0.30% (0.18-0.49)
Verrucomicrobiae (P: Verrucomicrobia)	0.10% (0.0-1.48)	0.43% (0.00-2.02)	0.02% (0.00-0.70)
**Families**			
Ruminococcaceae (P: Firmicutes; C: Clostridia; O: Clostridiales)	21.7% (16.2-26.2)	21.5% (16.2-25.2)	22.1% (17.1-27.7)
Bacteroidaceae (P: Bacteroidetes; C: Bacteroidia; O: Bacteroidales)	21.6% (12.4-28.8)	21.5% (13.0-28.9)	22.0% (12.1-28.7)
Lachnospiraceae (P: Firmicutes; C: Clostridia; O: Clostridiales)	9.10% (7.40-11.8)	10.7% (8.41-13.0)	8.01% (6.00-11.0)
Porphyromonadaceae (P: Bacteroidetes; C: Bacteroidia; O: Bacteroidales)	5.86% (4.04-8.14)	6.11% (4.30-8.90)	5.70% (3.89-7.85)
Rikenellaceae (P: Bacteroidetes; C: Bacteroidia; O: Bacteroidales)	4.77% (2.88-7.47)	4.01% (2.03-5.46)	5.53% (3.32-9.79)
Sutterellaceae (P: Proteobacteria; C: Betaproteobacteria; Burkholderiales)	2.78% (1.09-4.41)	2.47% (0.76-4.00)	3.18% (1.31-4.65)
Prevotellaceae (P: Bacteroidetes; C: Bacteroidia; O: Bacteroidales)	2.29% (0.12-17.5)	2.97% (0.09-15.8)	1.66% (0.12-19.7)
Desulfovibrionaceae (P: Proteobacteria; C: Deltaproteobacteria; O: Desulfovibrionales)	0.68% (0.35-1.18)	0.57% (0.32-0.83)	0.83% (0.47-1.58)
Acidaminococcaceae (P: Firmicutes; C: Negativicutes; O: Selenomonadales)	0.42% (0.02-1.23)	0.49% (0.03-1.38)	0.36% (0.01-0.97)
Coriobacteriaceae (P: Actinobacteria; C: Actinobacteria; O: Coriobacteriales)	0.34% (0.17-0.90)	0.28% (0.15-0.46)	0.49% (0.22-1.28)
Erysipelotrichaceae (P: Firmicutes; C: Erysipelotrichia; O: Erysipelotrichales)	0.33% (0.19-0.56)	0.33% (0.18-0.52)	0.32% (0.19-0.59)
Bifidobacteriaceae (P: Actinobacteria; C: Actinobacteria; O:Bifidobacteriales)	0.31% (0.08-1.16)	0.25% (0.08-0.79)	0.36% (0.06-1.75)
Enterobacteriaceae (P: Proteobacteria; C: Gammaproteobacteria; O: Enterobacteriales)	0.19% (0.05-0.76)	0.25% (0.05-1.40)	0.15% (0.04-0.62)
**Genera**			
Bacteroides (P: Bacteroidetes; C: Bacteroidia; O: Bacteroidales; F: Bacteroidaceae)	22.6% (13.0-29.5)	21.5% (13.5-27.2)	23.3% (12.2-31.7)
Faecalibacterium (P: Firmicutes; C: Clostridia; O: Clostridiales; F: Ruminococcaceae)	7.76% (4.45-10.9)	7.40% (3.87-10.4)	8.17% (5.94-11.4)
Alistipes (P: Bacteroidetes; C: Bacteroidia; O: Bacteroidales; F: Rikenellaceae)	4.44% (2.70-7.21)	3.91% (1.89-5.23)	5.28% (3.14-9.51)
Parabacteroides (P: Bacteroidetes; C: Bacteroidia; O: Bacteroidales; F: Porphyromonadaceae)	2.53% (1.66-4.09)	2.55% (1.72-4.16)	2.41% (1.47-4.01)
Oscillibacter (P: Firmicutes; C: Clostridia; O: Clostridiales; F: Ruminococcaceae)	2.30% (1.39-3.61)	2.06% (1.23-2.97)	2.76% (1.56-4.07)
Barnesiella (P: Bacteroidetes; C: Bacteroidia; O: Bacteroidales; F: Porphyromonadaceae)	1.28% (0.49-2.40)	1.50% (0.60-2.40)	1.11% (0.43-2.42)

Lachnospiraceae incertae sedis (P: Firmicutes; C: Clostridia; O: Clostridiales; F: Lachnospiraceae)	0.88% (0.52-1.60)	1.05% (0.53-1.72)	1.11% (0.43-2.42)
Roseburia(P: Firmicutes; C: Clostridia; O: Clostridiales; F: Lachnospiraceae)	0.76% (0.35-1.39)	0.67% (0.25-1.37)	0.87% (0.44-1.44)
Bilophila (P: Proteobacteria; C: Deltaproteobacteria; O: Desulfovibrionales; F: Desulfovibrionaceae)	0.46% (0.24-0.78)	0.49% (0.25-0.95)	0.44% (0.23-0.70)
Butyricimonas (P: Bacteroidetes; C: Bacteroidia; O: Bacteroidales; F: Porphyromonadaceae)	0.45% (0.19-0.78)	0.40% (0.11-0.69)	0.51% (0.25-0.81)
Odoribacter (P: Bacteroidetes; C: Bacteroidia; O: Bacteroidales; F: Porphyromonadaceae)	0.38% (0.21-0.60)	0.45% (0.31-0.66)	0.27% (0.15-0.48)
Ruminococcus (P: Firmicutes; C: Clostridia; O: Clostridiales; F: Ruminococcaceae)	0.35% (0.11-0.61)	0.37% (0.16-0.90)	0.30% (0.10-0.64)
Parasutterella (P: Proteobacteria; C: Betaproteobacteria; O: Burkholderiales; F: Sutterellaceae)	0.31% (0.10-1.47)	0.30% (0.10-1.44)	0.31% (0.09-1.49)
Sutterella (P: Proteobacteria; C: Betaproteobacteria; O: Burkholderiales; F: Sutterellaceae)	0.30% (0.00-3.15)	0.22% (0.00-2.51)	0.68% (0.00-3.51)
Blautia (P: Firmicutes; C: Clostridia; O: Clostridiales; F: Lachnospiraceae)	0.28% (0.17-0.54)	0.31% (0.18-0.64)	0.27% (0.16-0.45)
Bifidobacterium (P: Actinobacteria; C: Actinobacteria; O: Bifidobacteriales; F: Bifidobacteriaceae)	0.26% (0.07-0.97)	0.33% (0.08-1.55)	0.21% (0.05-0.72)
Clostridium XVIa (P: Firmicutes; C: Clostridia; O: Clostridiales; F: Lachnospiraceae)	0.24% (0.09-0.49)	0.15% (0.05-0.37)	0.35% (0.11-0.63)
Dorea (P: Firmicutes; C: Clostridia; O: Clostridiales; F: Lachnospiraceae)	0.12% (0.05-0.24)	0.14% (0.05-0.27)	0.10% (0.06-0.24)
Escherichia Shigella (P: Proteobacteria; C: Gammaproteobacteria; O: Enterobacteriales; F: Enterobacteriaceae)	0.10% (0.02-0.32)	0.08% (0.02-0.27)	0.06% (0.02-0.37)
Akkermansia (P: Verrucomicrobia; C: Verrucomicrobiae; O: Verrucomicrobiales; F: Verrucomicrobiaceae)	0.10% (0.01-1.48)	0.43% (0.01-2.02)	0.03% (0.01-0.70)
**Species**			
Faecalibacterium prausnitzii (P: Firmicutes; C: Clostridia; O: Clostridiales; F:Ruminococcaceae; G: Faecalibacterium)	3.96% (2.12-5.70)	3.52% (1.77-5.47)	4.35% (2.32-5.83)
Alistipes putredinis (P: Bacteroidetes; C: Bacteroidia; O: Bacteroidales; F: Rikenellaceae; G: Alistipes)	1.91% (0.88-3.20)	1.60% (0.75-2.62)	2.39% (1.12-3.90)
Bacteroides vulgatus (P: Bacteroidetes; C: Bacteroidia; O: Bacteroidales; F: Bacteroidaceae; G: Bacteroides)	1.87% (0.74-5.10)	1.86% (0.65-4.82)	2.13% (0.88-5.53)
Bacteroides sp. WA1 (P: Bacteroidetes; C: Bacteroidia; O: Bacteroidales; F: Bacteroidaceae; G: Bacteroides)	1.58% (0.46 2.82)	1.30% (0.39-2.46)	1.77% (0.51-4.07)
Ruminococcaceae bacterium LM158 (P: Firmicutes; C: Clostridia; O: Clostridiales; F:Ruminococcaceae; G: Oscillibacter)	0.76% (0.18-1.40)	0.66% (0.18-1.20)	0.86% (0.18-1.57)
Parabacteroides distasonis (P: Bacteroidetes; C: Bacteroidia; O: Bacteroidales; F: Porphyromonadaceae; G: Parabacteroides)	0.75% (0.36-1.29)	0.70% (0.35-1.26)	0.76% (0.39-1.67)
Parabacteroides merdae (P: Bacteroidetes; C: Bacteroidia; O: Bacteroidales; F: Porphyromonadaceae; G: Parabacteroides)	0.65% (0.17-1.32)	0.78% (0.25-1.39)	0.59% (0.16-1.10)
Bacteroides vulgatus ATCC 8482 (P: Bacteroidetes; C: Bacteroidia; O: Bacteroidales; F: Bacteroidaceae; G: Bacteroides)	0.47% (0.11-1.22)	0.60% (0.18-1.36)	0.31% (0.06-1.20)
Oscillospiraceae bacterium AIP 1035.11 (P: Firmicutes; C: Clostridia; O: Clostridiales; F:Ruminococcaceae; G: Oscillibacter)	0.41% (0.17-0.73)	0.23% (0.08-0.47)	0.61% (0.28-1.13)
Bilophila wadsworthia (P: Proteobacteria; C: Deltaproteobacteria; O: Desulfovibrionales; F: Desulfovibrionaceae; G: Bilophila)	0.32% (0.13-0.56)	0.23% (0.12-0.40)	0.39% (0.15-0.76)
Bacteroides uniformis (P: Bacteroidetes; C: Bacteroidia; O: Bacteroidales; F: Bacteroidaceae; G: Bacteroides)	0.25% (0.09-0.51)	0.20% (0.08-0.39)	0.32% (0.11-0.60)
Barnesiella sp. EBA4-14 (P: Bacteroidetes; C: Bacteroidia; O: Bacteroidales; F: Porphyromonadaceae; G: Barnesiella)	0.22% (0.08-0.82)	0.13% (0.06-0.41)	0.16% (0.04-0.40)
Bacteroides caccae (P: Bacteroidetes; C: Bacteroidia; O: Bacteroidales; F: Bacteroidaceae; G: Bacteroides)	0.21% (0.05-0.51)	0.32% (0.15-0.61)	0.14% (0.00-0.37)
Barnesiella intestinihominis (T) (P: Bacteroidetes; C: Bacteroidia; O: Bacteroidales; F: Porphyromonadaceae; G: Barnesiella)	0.21% (0.06-0.60)	0.27% (0.08-0.52)	0.18% (0.04-0.75)
Parasutterella excrementihominis (T) (P: Proteobacteria; C: Betaproteobacteria; O: Burkholderiales; F: Sutterellaceae; G: Parasutterella)	0.21% (0.04-1.13)	0.23% (0.07-1.13)	0.16% (0.03-1.16)
Bacteroides sp. S-18 (P: Bacteroidetes; C: Bacteroidia; O: Bacteroidales; F: Bacteroidaceae; G: Bacteroides)	0.18% (0.01-0.92)	0.18% (0.01-0.85)	0.14% (0.01-1.10)
Odoribacter splanchnicus (P: Bacteroidetes; C: Bacteroidia; O: Bacteroidales; F: Porphyromonadaceae; G: Odoribacter)	0.18% (0.90-0.33)	0.24% (0.12-0.42)	0.15% (0.07-0.26)
Alistipes sp. NML05A004 (P: Bacteroidetes; C: Bacteroidia; O: Bacteroidales; F: Rikenellaceae; G: Alistipes)	0.14% (0.04-0.36)	0.11% (0.04-0.24)	0.22% (0.04-0.40)
Ruminococcus bromii (P: Firmicutes; C: Clostridia; O: Clostridiales; F:Ruminococcaceae; G:Ruminococcus)	0.13% (0.01-0.35)	0.13% (0.01-0.31)	0.12% (0.01-0.40)
Bacteroides sp. ANH 2438 (P: Bacteroidetes; C: Bacteroidia; O: Bacteroidales; F: Rikenellaceae; G: Alistipes)	0.12% (0.03-0.34)	0.12% (0.01-0.30)	0.13% (0.03-0.50)
Akkermansia Muciniphila (P:Verrucomicrobia; C: Verrucomicrobiae; O: Verrucomicrobiales; F: Verrucomicrobiaceae; G:Akkermansia)	0.10% (0.01-1.32)	0.38% (0.01-1.71)	0.02% (0.00-0.63)
Bifidobacterium Longum (P: Actinobacteria; C: Actinobacteria; O: Bifidobacteriales; F: Bifidobacteriaceae; G: Bifidobacterium)	0.10% (0.01-0.22)	0.08% (0.01-0.29)	0.04% (0.00-0.19)
Eubacterium eligens ATCC 27750 (P: Firmicutes; C: Clostridia; O: Clostridiales; F: Lachnospiraceae; G: Lachnospiracea incertae sedis)	0.10% (0.02-0.33)	0.12% (0.02-0.30)	0.10% (0.02-0.35)
Lachnospira pectinoschiza (P: Firmicutes; C: Clostridia; O: Clostridiales; F: Lachnospiraceae; G: Lachnospiracea incertae sedis)	0.10% (0.03-0.20)	0.10% (0.02-0.21)	0.10% (0.05-0.19)
