# Supplementary material for: Role of Gut Microbiota in Overweight Susceptibility in an Adult Population in Italy
Source: Nutrients. 2023 Jun 21;15(13):2834. doi: 10.3390/nu15132834 (PMC10343630; doi:10.3390/nu15132834)
Supplement: Supplementary file 1 [file nutrients-15-02834-s001.zip › Table S2.pdf]

Table S2. Univariable and multivariable linear regression analyses. Dependent variable: Waist Circumference (WC)

**Dependent variable: WC**	**Model 1/Crude (beta, P)**	**Model 2 (beta, P)**	**Model 3 (beta, P)**	**Model 4 (beta,P)**	**Model 5 (beta,P)**	**Model 6 (beta, P)**
Firmicutes/Bacteroidetes Ratio	-0.23 (0.003)	-0.21 (<0.001)	-0.16 (0.012)	-0.14 (0.033)	-0.14 (0.037)	-0.09 (0.161)
Age		0.24 (<0.001)	0.25 (<0.001)	0.24 (<0.001)	0.24 (<0.001)	0.268 (<0.001)
Male, n (%)		-0.59 (<0.001)	-0.57 (<0.001)	-0.57 (<0.001)	-0.57 (<0.001)	-0.56 (<0.001)
Cardiovascular diseases, n (%)		0.13 (0.020)	0.13 (0.018)	0.14 (0.016)	0.14 (0.016)	0.12 (0.038)
Thyroid diseases, n (%)		0.04 (0.540)	0.05 (0.354)	0.05 (0.370)	0.05 (0.392)	0.07 (0.238)
Shannon Index			-0.05 (0.448)	-0.04 (0.514)	-0.04 (0.511)	-0.03 (0.666)
Phylum *Actinobacteria*			-0.004 (0.948)	0.02 (0.931)	0.02 (0.923)	0.05 (0.879)
Phylum *Proteobacteria*			0.05 (0.344)	0.04 (0.603)	0.03 (0.619)	0.04 (0.619)
Phylum *Verrucomicrobia*			-0.10 (0.079)	-0.31 (0.509)	-0.29 (0.546)	-0.13 (0.775)
Class *Actinobacteria*				0.03 (0.916)	0.03 (0.924)	-0.021 (0.983)
Class *Verrucomicrobia*				-0.01 (0.998)	-0.09 (0.984)	0.05 (0.958)
Class *Betaproteobacteria*				0.02 (0.857)	0.02 (0.854)	0.01 (0.910)
Genus *Bifidobacterium*				-0.07 (0.447)	-0.07 (0.460)	-0.03 (0.731)
Genus *Akkermansia*				0.22 (0.961)	-0.163 (0.972)	-0.59 (0.897)
Genus *Sutterella*				0.02 (0.786)	0.02 (0.780)	0.04 (0.581)
Species *Akkermansia Muciniphila*					0.262 (0.723)	0.37 (0.608)
Yogurt intake						-0.06 (0.298)
Whole foods intake						-0.09 (0.122)
Fruits and vegetales intake						-0.14 (0.015)

Data are expressed as linear regression coefficients and P values.
